# Genomic and Metabolomic Analyses of Natural Products in *Nodularia spumigena* Isolated from a Shrimp Culture Pond

**DOI:** 10.3390/toxins12030141

**Published:** 2020-02-25

**Authors:** Rafael Vicentini Popin, Endrews Delbaje, Vinicius Augusto Carvalho de Abreu, Janaina Rigonato, Felipe Augusto Dörr, Ernani Pinto, Kaarina Sivonen, Marli Fatima Fiore

**Affiliations:** 1 Center for Nuclear Energy in Agriculture, University of São Paulo, Avenida Centenário 303, Piracicaba 13400-970, São Paulo, Brazil; rafael.popin@helsinki.fi (R.V.P.); endrews.delbaje@gmail.com (E.D.); vini.abreu@gmail.com (V.A.C.d.A.); jana_rigonato@yahoo.com.br (J.R.); ernani@usp.br (E.P.); 2 Department of Microbiology, University of Helsinki, Viikinkaari 9, FI-00014 Helsinki, Finland; kaarina.sivonen@helsinki.fi; 3 Institute of Exact and Natural Sciences, Federal University of Pará, Rua Augusto Corrêa 1, Belém 66075-10, Pará, Brazil; 4 Faculty of Pharmaceutical Sciences, University of São Paulo, Avenida Professor Lineu Prestes, São Paulo 05508-000, São Paulo, Brazil; fadorr@usp.br

**Keywords:** cyanobacteria, gene cluster, mass spectrometry, cyanotoxins, terpenes, water quality

## Abstract

The bloom-forming cyanobacterium *Nodularia spumigena* CENA596 encodes the biosynthetic gene clusters (BGCs) of the known natural products nodularins, spumigins, anabaenopeptins/namalides, aeruginosins, mycosporin-like amino acids, and scytonemin, along with the terpenoid geosmin. Targeted metabolomics confirmed the production of these metabolic compounds, except for the alkaloid scytonemin. Genome mining of *N. spumigena* CENA596 and its three closely related *Nodularia* strains—two planktonic strains from the Baltic Sea and one benthic strain from Japanese marine sediment—revealed that the number of BGCs in planktonic strains was higher than in benthic one. Geosmin—a volatile compound with unpleasant taste and odor—was unique to the Brazilian strain CENA596. Automatic annotation of the genomes using subsystems technology revealed a related number of coding sequences and functional roles. Orthologs from the *Nodularia* genomes are involved in the primary and secondary metabolisms. Phylogenomic analysis of *N. spumigena* CENA596 based on 120 conserved protein sequences positioned this strain close to the Baltic *Nodularia*. Phylogeny of the 16S rRNA genes separated the Brazilian CENA596 strain from those of the Baltic Sea, despite their high sequence identities (99% identity, 100% coverage). The comparative analysis among planktic *Nodularia* strains showed that their genomes were considerably similar despite their geographically distant origin.

## 1. Introduction

Shrimp farming in tropical and subtropical coastal areas is a growing activity due to market demand for high-quality protein intake [1]. Water quality management is of primary consideration in any shrimp farming, as its degradation is detrimental to shrimp growth, survival, market price, and production costs [2,3,4]. In general, shrimp farming is developed in constructed ponds near estuaries and lagoons along the coastline. Coastal waters are a common water source for shrimp ponds on most farms, either used directly or pumped via coastal lagoons [5]. Water nutrient over-enrichment in the ponds due to fertilizer use, shrimp excretions, and unconsumed aquafeeds ultimately lead to cyanobacterial blooms [6,7,8,9].

Several bloom-forming cyanobacteria are capable of producing toxins that are highly harmful to many eukaryotic organisms. *Nodularia spumigena*, a brackish water planktonic heterocytous cyanobacterium, has been responsible for major blooms along ocean coastlines, estuaries, brackish water basins, and saline lakes of the European, Australian/Oceania, and African continents [10,11,12,13,14,15,16,17,18,19]. The presence of a *N. spumigena* bloom was reported in saline lakes and ponds on the North and South American continents, in the United States [20,21], Mexico [22,23], Argentina [24], and Uruguay [25]. Despite being a frequent phenomenon worldwide, most studies with *N. spumigena* forming blooms have been reported in the Baltic Sea [12,26,27]. Toxicity is the major concern regarding the blooms of this species, which may cause human and animal poisonings [10,14,16,28,29,30,31]. For example, shrimp mortality in culture ponds was reported in 2010 during a *N. spumigena* bloom in Brazil [32,33].

*N. spumigena* produces the hepatotoxin nodularin (NOD) [34], a cyclic pentapeptide with a similar chemical structure to microcystin. NOD acts as an inhibitor of the serine/threonine protein phosphatases family, particularly phosphatases type 1 (PP1) and 2A (PP2A) of eukaryotic cells [35,36], and is a suspected carcinogen and tumor promoter [37]. Due to the persistence of NOD in the environment and possible processes of bioaccumulation in organisms, economic problems along with harm to animals and humans may occur in areas with blooms of this cyanobacterium [38,39,40,41]. Besides NOD, *N. spumigena* strains are known to produce protease inhibitors such as anabaenoeptins (APT), spumigins (SPU), and aeruginosins (AER) [42,43,44]. Furthermore, several cyanobacteria synthesize the terpenoid compounds geosmin and/or 2-methylisoborneol (MIB), which are volatile metabolites with unpleasant taste and odor that cause additional costs for water utilities and the loss of market demands in the aquaculture industry [45].

The first whole-genome *N. spumigena* sequence originated from the NOD-producing strain CCY9414 isolated from the southern Baltic Sea [46]. Subsequently, the Brazilian NOD-producing strain CENA596 isolated from a shrimp pond was sequenced [47]. Recently, another genome of NOD-producing *N. spumigena* from the northern Baltic Sea was reported [48,49], and a draft genome of a *Nodularia* sp. from a tidal flat sediment sample from Yatsu Tidal Flat, Narashino, Chiba, Japan, was deposited in the NCBI database. In this study, we used genome-guided approaches to investigate secondary metabolite pathways in the strain CENA596 isolated from a shrimp pond and its differences compared to other *Nodularia* genomes from distinct isolation sources. Furthermore, metabolomic analyses targeting the secondary metabolite gene clusters found in the *N. spumigena* CENA596 genome were performed by liquid chromatography–high-resolution quadrupole time-of-flight mass spectrometry (LC-HR-QTOF).

## 2. Results

### 2.1. Biosynthetic Potential

**Figure 1:**
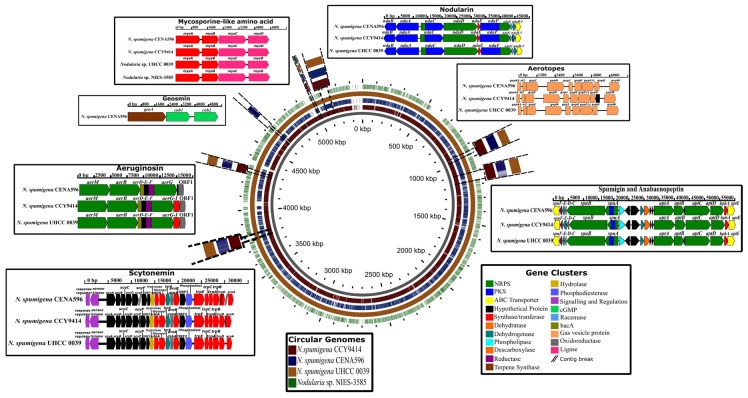
Blast atlas analysis of *Nodularia spumigena* CENA596, CCY9414, and UHCC 0039, and *Nodularia* sp. NIES-3585. The genome of the strain *N. spumigena* UHCC 0039 (a complete genome) was used as a reference to indicate the location of the gene clusters responsible for the biosynthesis of nodularin, anabaenopeptin, spumigin, aeruginosin, geosmin, gas vesicles, mycosporine-like amino acids, and scytonemin.

The number of metabolic pathways predicted by the antiSMASH server revealed a vast and distinctive yield of potential natural products in the genomes of the genus *Nodularia*, mainly in strains of a planktonic way of life (Table S1). Based on already known metabolic pathways, 13 natural product biosynthetic gene clusters were predicted in the genome of CENA596: two containing NRPSs, two containing polyketide synthases (PKSs), three containing hybrid NRPS and PKS (NRPS/PKS), two terpene metabolic gene clusters, two encoding ribosomally synthesized and post-translationally modified peptides (RIPPs), and two gene clusters not classified in any specific type. These gene clusters are related to the biosynthesis of NOD (*nda*), APT (*apt*), SPU (*spu*), AER (*aer*), geosmin (*geo*), aerotopes (*gvp*)*,* mycosporine-like amino acids (MAAs; *mys*), and scytonemin (SCY; *scy*) (Figure 1; Table S2). Gene clusters *nda*, *apt*, *spu*, and *aer* have already been identified in the CCY9414 genome, whereas we show the presence of gene clusters *mys*, *scy*, and *gvp* in this study. The strain UHCC 0039 genome encodes all analyzed gene clusters except *geo*. On the other hand, only *mys* was found in strain NIES-3585. We also analyzed the adenylation domain-binding pockets in *nda*, *spu*, *apt*, and *aer* of the three planktonic strains and showed them to be highly conserved (Table S3).

Gene cluster *nda* in the *N. spumigena* strains had a similar organizational structure, except for the presence of an open reading frame (ORF) between the *ndaH* and *ndaG* genes in the genome of the CCY9414 strain. Gene clusters *spu* and *apt*—encoded in the *N. spumigena* genomes—were separated by ~12 kilobase-pairs (kbp). Strain CENA596 showed an ORF encoding a hypothetical protein between *aptB* and *aptC*, and this gene is absent in the other strains. The *aer* gene cluster showed nearly the same organization in strains CENA596, CCY9414, and UHCC 0039, except for gene *aerI*, which was absent in the strain CENA596 and was replaced by an ORF encoding a hypothetical protein. The *geo* gene cluster was observed solely in the genome of the CENA596 strain and comprised a terpene synthase and two cyclic nucleotide-binding proteins. Gene cluster *gvp* in the CENA596 and UHCC 0039 strains showed an identical organization, whereas an ORF was found between genes *gvpG* and *gvpW* in strain CCY9414. On the other hand, *mys* showed to be highly conserved in the four strains. Last, the *scy* gene cluster was only found in the planktonic *N. spumigena* strains and was broken into four contigs in the Brazilian CENA596. All gene clusters showed slight differences in gene lengths and sequences (See Table S3).

### 2.2. Metabolomics

CENA596 produced two variants of NOD (NOD and [D-Asp^1^]NOD), SPU (D and F), and AER (NAL2 and NOL3) (File S1); compounds which were previously identified in CCY9414 and UHCC 0039 [42,44,50] (Table 1). However, the Baltic strains produced several other variants of SPU and AER that were not identified in CENA596. Before this study, the production of namalides (B and C) had not yet been reported in *N. spumigena*.

### 2.3. General Features of Nodularia Genomes 

**Figure 2:**
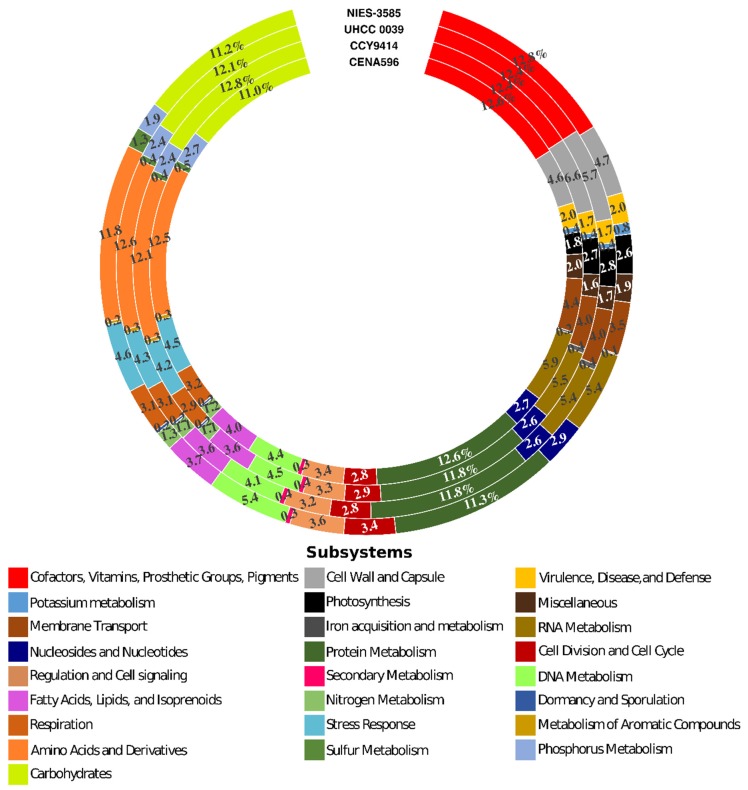
RAST Subsystem annotation of the *Nodularia spumigena* strains CENA596, CCY9414, and UHCC 0039, and *Nodularia* sp. NIES-3585 genomes. The values represent the percentage of classified sequences in the determined subsystem.

The comparison of CENA596, CCY9414, UHCC 0039, and NIES-3585 genomes assembly statistics revealed resemblances among them (Table 2). Although the CENA596 genome is still in draft status, the total genome size is considerably close to those of CCY9414 and UHCC 0039. Differently, the draft assembly of NIES-3585 showed the largest genome. The GC% content of the strains was similar (≅41.2%), but due to the number of scaffolds, CENA596 showed the lowest N50. The subsystem annotation identified 31–34% of the coding sequences from the four genomes. These sequences were mainly involved in cellular primary metabolism, such as the biosynthesis of vitamins, cofactors, pigments, and the metabolism of proteins, DNA, RNA, amino acids, and carbohydrates, photosynthesis, and respiration (Figure 2). The remaining 66–69% of all the genes were not classified in any subsystems.

**Figure 3:**
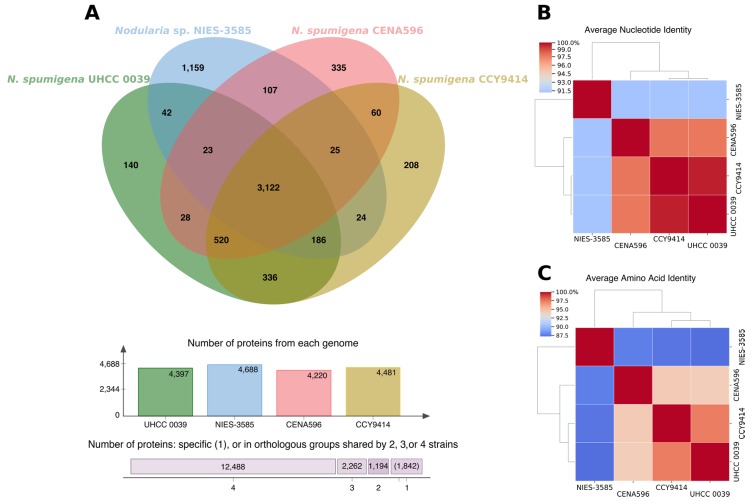
Comparative genome analyses of the *Nodularia spumigena* CENA596, CCY9414, and UHCC 0039, and *Nodularia* sp. NIES-3585. (**A**) Analysis of homologous proteins, (**B**) average nucleotide identity heatmap, and (**C**) average amino acid identity heatmap.

Approximately 74% of the protein encoded in the genomes of CENA596, 70% of CCY9414, 71% of UHCC 0039, and 67% of NIES-3585 were orthologs in the four genomes (3122 proteins) (Figure 3A). The percentage of proteins exclusively encoded in each genome (paralogous and singletons) was approximately 8%, 5%, 3%, and 25% for CENA596, CCY9414, UHCC 0039, and NIES-3585, respectively (19.43% of the total). The remaining 18% of the proteins from CENA596, 25% of CCY9414, 26% of UHCC 0039, and 8% of NIES-3585 were orthologs in two or three genomes (10.36% of the total). In terms of the total number of proteins (17,786), 12,488 (or 70.21%) were orthologs in the four genomes, 1842 (or 19.43%) were paralogues and singletons, and 3456 were orthologs in two or three genomes.

The automatic annotation performed with MG-RAST showed that the ortholog genes in the four genomes were involved in a wide range of functions such as primary metabolism (photosynthesis, respiration, metabolism of vitamin, cofactors, etc.) stress responses, secondary metabolism, and other cellular processes (membrane transport, regulation, and cell signaling, and cell division and cycle) (Figure S1A). The specific genes of each strain were classified in equally diverse functions (Figure S1B–E).

Strain CENA596 showed average nucleotide identity (ANI)/average amino acid identity (AAI) of 97.76%/94.45% with CCY9414 and 97.75%/94.32% with UHCC 0039 (Figure 3B,C, respectively). The two Baltic strains were considerably similar (99.64%/97.36%, respectively), whereas the Japanese NIES-3585 had smaller identities (91.15%/87.39%, 91.20%/87.69%, and 91.21%/87.14% to CENA596, CCY9414, and UHCC 0039, respectively).

### 2.4. Phylogenomic Analysis

**Figure 4:**
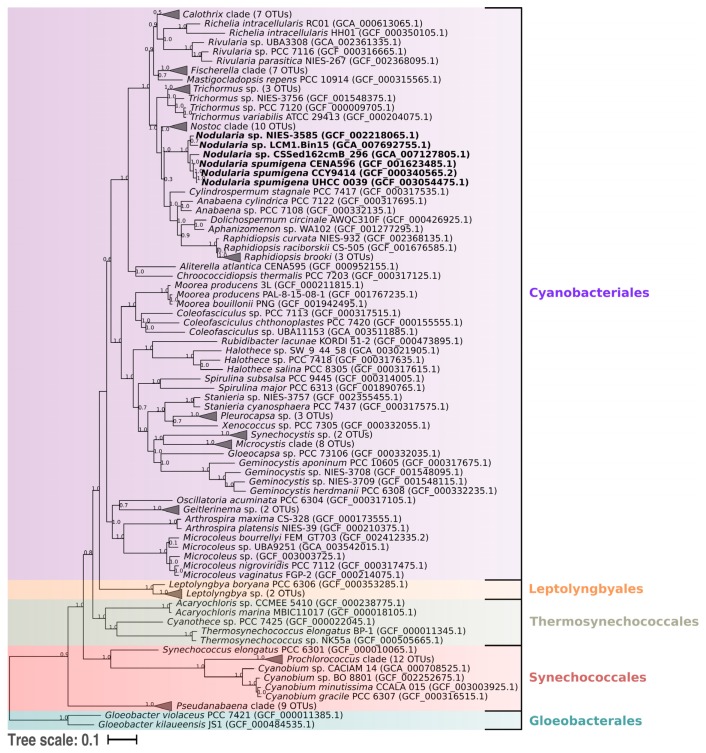
Maximum Likelihood phylogenomic tree based on 120 conserved proteins in cyanobacterial genomes. The strains *Nodularia spumigena* CENA596, CCY9414, and UHCC 0039, and *Nodularia* sp. NIES-3585 are shown in bold. Accession numbers of the sequences are presented in parentheses and GTDB taxonomy of the clades are indicated by different colors.

The phylogenomic tree grouped the strains within a clade containing other cyanobacteria belonging to the order Nostocales (Figure 4). The two evaluated Baltic strains formed a clade that shared a common ancestor with the Brazilian strain. The metagenome-assembled genome (MAG) CSSed162cmB_296 isolated from a Russian soda lake was positioned next to these three *Nodularia* strains. A sister clade comprised the Japanese strain and the Canadian MAG LCM1.Bin15.

**Figure 5:**
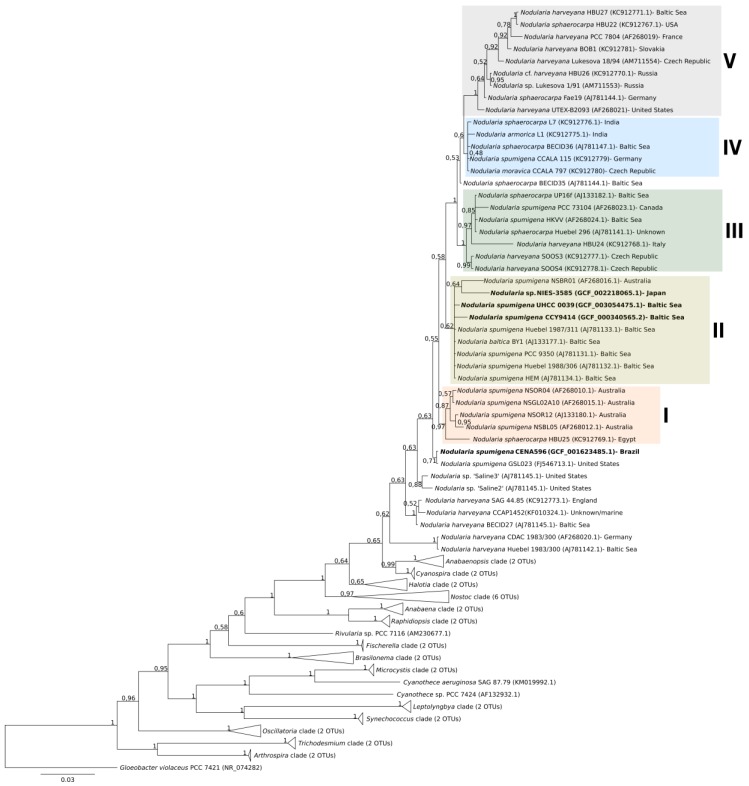
Bayesian inference tree based on the 16S rRNA genes from 81 cyanobacterial strains constructed using 1000 bootstrap replicates. The origin of *Nodularia* strains is presented and the accession number of the sequences is shown in parentheses. Strains *Nodularia spumigena* CENA596, CCY9414, and UHCC 0039, and *Nodularia* sp. NIES-3585 are highlighted in bold. Subgroups of strains are presented in different colors.

The 16S rRNA gene sequences showed high identities among each other (99%; data not provided), but the strains grouped in distinct clades in the phylogenetic tree (Figure 5). The Brazilian CENA596 was allocated with the strain GSL023 isolated from the Great Salt Lake, UT, USA. This clade was closely related to *N. spumigena* strains from Australian brackish waters (Subclade I). The sequences of seven *N. spumigena* strains from the Baltic Sea, including CCY9414 and UHCC 0039, were positioned together in Subclade II. The Japanese NIES-3585 and an Australian *N. spumigena* strain (NSBR01) were positioned close to these Baltic strains. *Nodularia* Subclades III, IV, and V included mixed strains from various species and geographical origins.

## 3. Discussion

Cyanobacteria are known for their abundance and capability of exploring a wide range of environments [53]. Strain CENA596 was isolated from shrimp production ponds in the southern region of Brazil [47]. The crustaceans are cultivated using the Biofloc Technology System, in which fertilizers are used to stimulate microbial action to mineralize and assimilate nutrients from the feeding and excretion of shrimp [54]. In this way, water can be used in several production cycles without needing to exchange it with the surrounding aquatic environment [55]. Although cyanobacteria naturally compete with algae in aquaculture tanks, cyanobacteria often dominate eutrophic tanks due to their higher ability to thrive with low dissolved oxygen, high temperature, and turbidity [56]. Moreover, the water salinities commonly used in shrimp ponds are optimal for *N. spumigena* growth [49,57,58,59]. Therefore, cyanobacteria may form blooms in these environments and negatively interfere with shrimp production [60]. Strain CCY9414 was isolated from samples collected near Bornholm, Denmark in 1996 [61]. During the summer, *N. spumigena* blooms overcome phosphorous limitation by degrading phosphonate and are favored in the Baltic Sea due to stable, stratified, and warm brackish water [46,48]. Strain UHCC 0039 (also known as AV1) was isolated from a water sample collected in 1987 from the open Gulf of Finland [48,49].

The application of genome sequencing and mining to analyze and classify BGCs in the *N. spumigena* CENA596 genome gave new insights on the potential of this strain to synthesize natural products. BGCs are the core organization of cyanobacterial biosynthetic pathways at the genome level and generally code for multidomain enzymes, such as PKSs and NRPSs, transporters, and tailoring enzymes (such as halogenases, oxidases, and cyclases) [62,63]. BGC expression is regulated at the transcriptional level, and regulatory mechanisms are frequently found flanking the BGC [64,65]. A search for gene clusters responsible for the synthesis of natural products in *N. spumigena* CENA596 revealed a similar genomic construction to that found in the genomes of the Baltic *N. spumigena* CCY9414 and UHCC 0039 strains. However, LC-HR-QTOF mass spectrometry analyses showed lower variant production of the nonribosomal peptides NOD, SPU, APT, and AER by the Brazilian strain than the Baltic strains [42,44,46,50]. The co-production of toxin (NOD) and odorous (geosmin) metabolites by *N. spumigena* CENA596 during bloom episodes in shrimp ponds raises concern for human and animal health issues along with economic losses. When consumed by humans and other animals, NOD may lead to hepatic structure modification and consequent tissue damage, organ failure, and hemorrhagic shock [66]. Bioaccumulation of this molecule in the environment is also of great concern [39,40,67,68].

Gene cluster *nda* was first identified in *N. spumigena* NSOR12 as being composed of nine genes 47kbp wide in total [69]. This gene cluster was later found in *N. spumigena* strain AV1 [70], followed by CCY9414 [46] and CENA596 [47]. The reason behind NOD production by cyanobacteria is unknown but probable functions have been proposed such as protection against oxidative and luminous stress, predation, and allelopathic competition [66,71]. We note that *nda* was absent in the Japanese benthic *Nodularia* sp. NIES-3585, which corroborates the report that benthic *Nodularia* strains are not NOD producers [72]. Therefore, the fact that benthic *Nodularia* does not produce NOD and the high conservation of *nda* among the three planktonic *N. spumigena* strains suggests an important adaptive advantage of NOD-producing strains for the exploration of the water surface.

The nonribosomal *spu* and *apt* found in the genome of *N. spumigena* CENA596 showed nearly identical gene organization as in *N. spumigena* CCY9414 and UHCC 0039 and *Sphaerospermopsis torques-reginae* ITEP-024. In these strains, *spu* and *apt* together are in a genome region separated only by a 12 kbp nucleotide sequence that is believed to encode biosynthetic enzymes of substrates involved in the synthesis of both metabolites [73]. SPU and APT are protease inhibitors [42,43]. Proteases are responsible for catalyzing the breakdown of proteins, and therefore are intimately related to cell cycle progression, cell proliferation, and death; DNA replication; tissue remodeling, homeostasis; wound healing; and the immune response in complex animals: the dysregulation of those enzymes may lead to cardiovascular and inflammatory diseases, cancer, osteoporosis, and neurological disorders [74]. Despite the presence of *apt* in the genome of CENA596, only namalides were identified by chemical analysis. The production of namalides by the APT peptide synthase pathway has been attributed to a module skipping event and has already been identified in *Nostoc* sp. CENA543 and *S. torques-reginae* ITEP-024 [75,76]. Namalides are cyclic tetrapeptides with an exogenous amino acid attached to the macrocycle by a urethane linkage and are structurally related to APT but lacking two amino acid residues [77]. These authors also reported the carboxypeptidase A inhibitory activity of namalide at submicromolar concentrations.

The *aer* of the Brazilian CENA596 showed an incomplete *aerI* gene acting as a transferase in the molecule biosynthesis. Events of gene inversion, deletion, rearrangements, fusion, and fission are frequent in bacterial genomes and are known as mechanisms of gene evolution, in which multi-domain proteins evolve [78,79]. The alteration found in gene *aerI* in CENA596 did not affect its expression, as two AER variants were detected by mass spectrometry analysis. Variability in the *aer* biosynthetic pathway has been associated with a varying production of AER analogs [80]. Many cyanobacterial genera produce AERs, and ~100 variants have been described [81]. In the genus *Nodularia*, *aer* was first described in *N. spumigena* CCY9414 [46]. AERs constitute a group of linear modified tetrapeptides, and since the first isolation [82], this class of natural products gained attention as protease inhibitors [83] and as potent biotoxins [84].

The ribosomal gene cluster involved in the synthesis of geosmin was identified only in the genome of *N. spumigena* CENA596, and gas chromatography analysis confirmed the production of this terpenoid. This is the second report of geosmin being produced by *N. spumigena* [85]. Geosmin is the dominant cause of the muddy and earthy taste and odor in drinking water and causes consumer complaints and economic losses in drinking water supplies and the fishing industry [45]. Although this odorous metabolite is produced by a wide range of organisms, including actinobacteria, proteobacteria, fungi, amoeba, and liverwort, cyanobacteria are considered to be the major source of geosmin in aquatic environments where photosynthetic growth is viable [86]. This terpene seems to be part of oxidative stress responses in fungi and may have the same function in cyanobacteria [87].

Gene clusters *mys* and *scy* found in the genomes of the four *Nodularia* and the three planktonic *N. spumigena*, respectively, are responsible for the synthesis of MAAs and SCY, which are strong UV-absorbing compounds. Their biosynthesis is a mechanism developed by certain cyanobacteria to avoid harmful biological effects of exposure to solar UV radiation [88]. In addition to their photoprotective function, these pigments are important antioxidants, compatible solutes, and intracellular nitrogen reservoirs that may show anti-inflammatory and antiproliferative activities without chemical toxicity [89,90]. Therefore, these alkaloid compounds have gained research attention due to potential economic importance as candidates for pharmaceutical and cosmetic applications. Mass spectrometry analysis of the Brazilian CENA596 detected the production of two MAAs—shinorine and porphyra-334—as previously identified [89]. SCY was not detected despite the presence of *scy*. The explanation for this may be that the genes are not being expressed or the production level is below the detection limit, as multiple environmental signals may reportedly act to determine the level of this pigment in various cyanobacteria species [91].

Cyanobacteria from genus *Nodularia* were divided into three ecological groups: planktonic, benthic, and terrestrial [92]. Phenotypic (presence or absence of aerotopes), genetic (16S rRNA gene sequences), and genomic features (short tandemly repeated repetitive sequence fingerprinting) differentiated aquatic lineages in the planktonic or benthic groups [72]. The genetic organization of the operons responsible for the synthesis of aerotope structure is susceptible to rearrangements that may lead to the loss of these structures [93]. The benthic NIES-3585 did not possess the *gvp*, and therefore, is expected to not form aerotopes, as commonly found in a strain of this lifestyle. Although planktonic *Nodularia* has been extensively studied due to the formation of toxic blooms, the existing studies on benthic and soil *Nodularia* studies are limited to a few areas [26,72,94]. This lack of studies may be related to them not being dominant in these biotopes and generally not producing nodularin, with the exception of the strain *N. sphaerocarpa* PCC7804, which was isolated from a benthonic mantle of a thermal spring in France and produces NOD and [L-Har^2^]NOD [95,96].

The subsystem annotation of the genomes revealed a similar pattern of functional annotation. This result point to a noticeable genetic similarity as found in a previous comparative study of the *N. spumigena* strains [49]. The majority of the orthologs from the four genomes remain unidentified, whereas those that were annotated were involved in key metabolic pathways necessary for the survival of the organisms but also adaptive advantages and the secondary metabolism. This set of orthologous genes originated during speciation [97,98]. Similarly to the orthologs, just a few of the specific genes were assigned to a function. Thus, the metabolic diversity of the strains is possibly linked to these unknown genes. 

The 16S rRNA gene phylogenetic analysis indicated that strain CENA596 was closely related to North American and Australian strains, whereas CCY9414 and UHCC 0039 were related to Baltic strains. The Japanese NIES-3585 allocated away from other benthic *Nodularia* but close to the Baltic *N. spumigena* strains. The phylogeny of this genus is problematic, as several species described based on their morphological characters did not present stable positions in phylogenetic trees [26,99]. Previous studies reported a clear separation between Baltic and Australian *N. spumigena*, although a limited number of sequences were analyzed [33,100,101]. Due to the inclusion of representatives from various geographical regions, our present results indicate that this geographical separation is not stable. Moreover, divergent subclades of Nodularia were previously proposed based on a limited number of 16S rRNA sequences [33,99]. The new subclades established here comprise strains from varied species and geographical regions and provide no clear separation based on these aspects. This result corroborates that the analysis of 16S rRNA is not an appropriate approach for studying the species categories and geographical origin of *Nodularia* [99].

16S rRNA gene-based phylogenies have been widely used for the identification of cyanobacteria [102], although the method has several limitations that may lead to poor resolutions in other bacterial taxa [103,104,105]. Therefore, the use of single-copy vertically inherited proteins has been proposed as a better approach to infer bacterial phylogeny [106,107]. In the specific case of cyanobacteria, analyses of 31 concatenated proteins provided robust results for the taxonomic study of these organisms [108,109,110]. The use of 120 conserved proteins has been recently proposed as a standard method for bacterial taxonomy and we introduced it to investigate the currently available *Nodularia* genomes and MAGs [111].

The approach used in our present work requires genome sequences of the organisms, only four of which are available for *Nodularia*. Although the two available MAGs of *Nodularia* were included, we were unable to conduct a robust analysis of the evolutionary relationship of this genus and therefore our analysis resembled a previous analysis constructed with 31 conserved proteins [49]. So far, no evident geographical difference among American, European, and Asian *Nodularia* can be pointed out. Both metagenomes are from soda lakes in the Northern Hemisphere. MAG CSSed162cmB_296 was grouped with the planktonic *N. spumigena* strains, whereas LCM1.Bin15 is positioned close to the benthic one. Future inclusion of new genomic sequences from distinct ecological niches will improve the taxonomy of *Nodularia* species and may establish subgroups containing various taxa.

## 4. Conclusions

Comparative analyses showed that the genomes of the Brazilian strain *N. spumigena* CENA596 and both the Baltic strains *N. spumigena* CCY9414 and UHCC 0039 are considerably more similar than the Japanese strain *Nodularia* sp. NIES-3585. As the function of most of the genes encoded in those genomes remains unclear, the biosynthetic diversity of the genus *Nodularia* was only evaluated based on known pathways, leaving potential BGCs to be further explored. So far, genome mining and chemical analyses of the available *N. spumigena* strains have already demonstrated the vast potential of this species to produce bioactive molecules, highlighting the need for monitoring harmful blooms in aquatic environments explored by human activities. The phylogeny of genus *Nodularia* still needs improvements, as the majority of genomic information available is from the species *N. spumigena*, which lacks representatives for most of the geographic regions. Future studies on the genus should use a phylogenomic approach based on the analysis of a large number of conserved proteins rather than the traditional 16S rRNA gene for a robust taxonomy. Last, generating new genomic information of isolates from other regions may contribute to better understanding of this genus, help prevent damages caused by toxic blooms, and enable the discovery of new natural products with pharmacological and biological activities.

## 5. Materials and Methods 

### 5.1. Cyanobacterial Genomes

The draft genome of the Brazilian strain *N. spumigena* CENA596 was published earlier [47] (accession number GCF_001623485.1). This strain was isolated from a *N. spumigena* bloom sample collected on 5 December 2013 in a shrimp production pond (32°12′19″S, 52°10′42″W) at the Marine Aquaculture Station of the Federal University of Rio Grande. The station is located on Cassino beach in the southwest Atlantic Ocean, Rio Grande municipality, Rio Grande do Sul state, Brazil. The strain *N. spumigena* CCY9414, with a possibly complete published genome, was isolated from a sample of brackish surface water collected in 1996 from the Baltic Sea near Bornholm [46,61] (accession number GCF_000340565.2). Strain *N. spumigena* UHCC 0039 (or *N. spumigena* AV1), with a complete genome, was isolated from a water sample collected in 1987 from the open Gulf of Finland [12,48,49] (accession number GCF_003054475.1). The draft genome of the Japanese strain *Nodularia* sp. NIES-3585 (accession number GCF_002218065.1) was included in this study because it showed a nucleotide identity of 99% to the *N. spumigena* CENA596 genome.

### 5.2. Natural Product Pathways in N. spumigena CENA596

Automatic annotation of secondary metabolite gene clusters in the analyzed genomes was performed using the antiSMASH server v4.0 [112]. Manual annotation and curation were then performed with the Artemis program v17.0.1 [113] and BLASTP (http://blast.ncbi.nlm.nih.gov/Blast.cgi?PAGE=Proteins). For that, protein sequences of known secondary metabolite pathways were compared with the GenBank protein database using the BLASTP tool to detect orthologs (*e*-value ≤ 1 × 10^−20^, identity ≥ 30%). The specificity of the adenylation domain of nonribosomal peptide synthetases (NRPSs) enzymes was predicted using the server NRPSpreditor2 [114].

### 5.3. Targeted Metabolomics

*N. spumigena* CENA596 was cultured in an F/2 liquid medium [115] without silica. Cultures were maintained at 22 ± 1 °C following a light–dark cycle of 14:10 h with fluorescent light (40–50 µmol photons∙m^−2^∙s^−1^) for 20 days. Fresh samples (200 mL) were vacuum-filtered on glass fiber (Sartorius GmbH, Germany) for cell concentration. Samples were extracted with 1 mL of MeOH:H_2_O 60/40 (*v*/*v*) at ultrasound probe (Sonic Ruptor 400, Omni, GA, USA) during 1 min on ice. After centrifugation for 10 min at 12,000× *g* (5804R, Eppendorf, Germany), the supernatant was separated and the extraction repeated. Combined supernatants were filtered (Millex 0.45 μm, Millipore, MA, USA) into vials. Analyses of NOD, SPU, APT, AER, mycosporine-like amino acids (MAA), and scytonemin (SCY) were performed on a Luna C18(2) column (150 × 2.1 mm, 3 μm; Phenomenex, State abbreviation, USA) using (A) 0.1% formic acid and (B) acetonitrile as the mobile phase (250 μL∙min^−1^ at 35 °C). The separation was obtained in a Shimadzu Prominence equipment (Kyoto, Japan) with a linear gradient from 5% to 90% B in 34 min (a total time of 45 min). High-resolution mass spectrometry data were acquired on a quadrupole/time-of-flight instrument (MicroTOF-QII, Bruker Daltonics, MA, USA) equipped with an electrospray source (ESI) operated in the positive ionization mode. Compound scanning was performed from *m/z* 100 to 1500 using a sodium acetate solution for mass calibration. Product ion spectra were acquired using argon as a collision gas under variable dissociation energies. Geosmin analysis was performed as described previously [45] using solid-phase microextraction and gas chromatography coupled to mass spectrometry (7890A/5975C, Agilent Technologies, CA, USA). The identification of compounds in CENA596 was based on their exact mass (<5 ppm) and their MS^2^ profile compared to previous spectra available in the literature (molecular data is shown in File S1). The terpenoid geosmin was identified based on its retention time and mass spectrum profile by GC-MS compared to an analytical standard and the NIST library.

### 5.4. Comparative Analyses

Genome assembly statistics were obtained using Assemblathon 2 [116]. A genome-wide comparison of the four *N. spumigena* and *Nodularia* sp. genomes was conducted using Blast Atlas analysis in GView Server v.3 [117]. Subsystems annotation was performed with the RAST server [51] and SEED tool [52]. The OrthoVenn server was used in the analysis of orthologous clusters [118] while MG-RAST v4.0.3 was used for the automatic annotation of the orthologs and specific proteins [119]. Heatmaps were estimated using the program GET_HOMOLOGUES [120,121] and generated using a seaborn v0.9 library script (https://github.com/mwaskom/seaborn/releases).

### 5.5. Phylogenomic Analysis

The phylogenomic placement was inferred with GTDB-Tk v0.3.2 (database release 89, https://github.com/Ecogenomics/GTDBTk) based on the Genome Taxonomy Database (GTDB), which is a recently proposed genomic-based taxonomy for Bacteria and Archaea [111]. The pipeline generates the tree through the identification and alignment of 120 bacterial single-copy conserved marker genes, then infers the phylogeny of the concatenated sequences using FastTree [122] with the WAG+GAMMA models and maximum likelihood algorithm. MAG of *Nodularia* publicly available in the NCBI was included in the analysis to expand the number of representatives of the genus.

The 16S ribosomal RNA (rRNA) gene tree was generated using an alignment performed with Muscle alignment in MEGA v10.0.5 (default parameters) of 81 cyanobacterial sequences [123]. The evolutionary model GTR+I+G best fitted the data set according to jModelTest v2.1.1 [124] and the Bayesian inference was constructed by MrBayes v3.2.6 with 5,000,000 generations [125]. 

## Figures and Tables

**Table 1:** Natural products identified in the *Nodularia spumigena* CENA596 and comparison to those previously identified in strains CCY9414 and UHCC 0039 (AV1) [42,44,50]. MW represents the molecular weight of the compounds. The subunits are the amino acids that compose the molecules. See also File S1 for further molecular data on the compounds identified in CENA596.

Natural Product		Structural Subunits						Strain		
Nodularin	MW	1	2	3	4	5	6	CENA596	CCY9414	UHCC0039
NOD	824	MeAsp	Arg	Adda	Glu	MeDhb	*	+	+	+
[D-Asp^1^] NOD	810	Asp	Arg	Adda	Glu	MeDhb	*	+	+	+
**Spumigin**	**MW**	**1**	**2**	**3**	**4**	**5**	**6**	**CENA596**	**CCY9414**	**UHCC0039**
A	612	Hpla	Hty	mPro	Argol	*	*	-	+	+
B1	626	Hpla	Hty	mPro	Arg	*	*	-	+	+
B2	626	Hpla	Hty	mPro	Arg	*	*	-	+	+
D	598	Hpla	Hty	Pro	Argol	*	*	+	+	+
E	610	Hpla	Hty	mPro	Argal	*	*	-	+	+
F	596	Hpla	Hty	Pro	Argal	*	*	+	+	+
G	596	Hpla	Hph	mPro	Argal	*	*	-	+	+
H	580	Hpla	Hph	Pro	Argal	*	*	-	+	+
582b	582	Hpla	Hty	mPro	Agm	*	*	-	-	+
**Anabaenopeptin**	**MW**	**1**	**2**	**3**	**4**	**5**	**6**	**CENA596**	**CCY9414**	**UHCC0039**
NP933	933	Phe	Lys	Val	Hty	MeHty	MetO	-	+	+
NPA	929	Ile	Lys	MetO2	Hph	MeHty	AcSer	-	+	+
NP915a	915	Ile	Lys	MetO	Hph	MeHty	Met	-	+	+
NPB	913	Ile	Lys	MetO	Hph	MeHty	AcSer	-	+	+
NP 839	839	Ile	Lys	Met	Hph	MeHph	Ser	-	+	+
NP 849	849	Ile	Lys	Val	Hph	MeHph	AcSer	-	-	+
NP 855a	855	Ile	Lys	Met	Hph	MeHty	Ser	-	+	+
NP 855b	855	Ile	Lys	MetO	Hph	MeHph	Ser	-	+	-
NP 881a	881	Ile	Lys	Met	Hph	MeHph	AcSer	-	+	+
NP 881b	881	Ile	Lys	Ile	Hph	MeHty	Met	-	+	+
NP 879	879	Ile	Lys	Ile	Hph	MeHty	AcSer	-	+	+
[Ser^6^] NPB	871	Ile	Lys	MetO	Hph	MeHty	Ser	-	+	+
NP 867	867	Ile	Lys	Val	Hph	MeHty	Met	-	-	+
NP 865	865	Ile	Lys	Val	Hph	MeHty	AcSer	-	-	+
NP 863	863	Ile	Lys	Ile	Hph	MeHph	Ser	-	-	+
NP 883a	883	Ile	Lys	Met	Hph	MeHph	Met	-	+	+
[Met^6^] NPC	899	Ile	Lys	Met	Hph	MeHty	Met	-	+	+
NPC	897	Ile	Lys	Met	Hph	MeHty	AcSer	-	+	+
[MeHph^5^] NPB	897	Ile	Lys	MetO	Hph	MeHph	AcSer	-	+	+
**Namalide**	**MW**	**1**	**2**	**3**	**4**	**5**	**6**	**CENA596**	**CCY9414**	**UHCC0039**
B	575	Ile	Lys	Ile/Leu	Hty	*	*	+	–	–
C	561	Ile	Lys	Val	Hty	*	*	+	–	–
**Aeruginosin**	**MW**	**1**	**2**	**3**	**4**	**5**	**6**	**CENA596**	**CCY9414**	**UHCC0039**
NAL1	558	Bu	Tyr	Choi	Argal	*	*	-	+	+
NAL2	586	Hex	Tyr	Choi	Argal	*	*	+	+	+
NAL3	614	Oct	Tyr	Choi	Argal	*	*	+	+	+
NOL1	532	Ac	Tyr	Choi	Argol	*	*	-	+	+
NOL2	560	Bu	Tyr	Choi	Argol	*	*	-	+	+
NOL3	588	Hex	Tyr	Choi	Argol	*	*	+	+	+
NOL4	616	Oct	Tyr	Choi	Argol	*	*	-	+	+
**Terpenes**	**MW**	**1**	**2**	**3**	**4**	**5**	**6**	**CENA596**	**CCY9414**	**UHCC0039**
Geosmin	*	*	*	*	*	*	*	+	-	-
**Mycosporine-like amino acids**	**MW**	**1**	**2**	**3**	**4**	**5**	**6**	**CENA596**	**CCY9414**	**UHCC0039**
Shinorine	332	Maa	Gly	Ser	*	*	*	+	-	-
Porphyra 334	346	Maa	Gly	Thr	*	*	*	+	-	-

MeAsp: Methylaspartate; Arg: Arginine; Adda: 3-amino-9-methoxy-2-6,8-trimethyl-10-phenyl-4,6-decadienoic acid; Glu: Glutamate; MeDhb: N-methyl-dehydrobutyryl;D-MeAsp: D-erythro-ß-methyl-aspartic acid; Asp: Aspartate; Hpla: 3-4-hydroxyphenyl lactic acid; Hty: Homotyrosine; mPro: 4-methyproline; Argol: Argininol; Pro: Proline; Argal: Argininal; Hph: Homophenylalanine; Agm: Agmatine; Phe: Phenylalanine; Lys: Lysine; Val: Valine; MeHty: Methyltyrosine; MetO: Methionine; Ile: Isoleucine; MetO2: Methionine sulfone; AcSer: Acetylserine; MetO: Methionine sulfoxide; Met: Methionine; Ser: Serine; MeHph: Methylhomophenylalanine; Bu: Butyric acid; Tyr: Tyrosine; Hex: Hexanoic acid; Choi: 2-carboxy-6-hydroxyoctahydroindole; Oct: Octanoic acid; Ac: Acetic Acid; Maa: Mycosporine; Gly: Glycine; *: Not applicable; +: Detection of the product; -: Absence of the product.

**Table 2:** Comparison of genome assembly statistics of *Nodularia spumigena* strains CENA596, CCY9414, and UHCC 0039, and *Nodularia* sp. NIES-3585. Subsystem statistics were obtained using the RAST server [51] and SEED tool [52].

Genome Statistics	*Nodularia spumigena*			*Nodularia* sp.
	CENA596	CCY9414	UHCC 0039	NIES-3585
Number of contigs	291	76	2	20
Number of scaffolds	-	1	-	4
Number of plasmids	-	-	1	-
Total size (bp)	5,189,679	5,462,271	5,386,612	5,773,538
Max scaffolds length (bp)	109,819	5,462,271	5,294,286	5,482,519
Min scaffolds length (bp)	526	5,462,271	92,326	58,866
Mean scaffold size (bp)	17,834	5,462,271	2,693,306	1,443,384
Median scaffold size (bp)	12,498	5,462,271	2,693,306	156,947
GC content (%)	41.2	41.19	41.2	41.2
N50 (bp)	32,474	5,462,271	5,294,286	1,022,420
Subsystem annotation statistics	-	-	-	-
Number of subsystems	370	378	383	387
Number of coding sequences	4907	5277	5207	5546
Coding sequences in subsystems	1595 (33%)	1704 (33%)	1727 (34%)	1700 (31%)
Coding sequences not in subsystems	3312 (67%)	3573 (67%)	3480 (66%)	3846 (69%)

## Supplementary Materials

The following are available online at https://www.mdpi.com/2072-6651/12/3/141/s1, Figure S1: Orthologs (**A**) and specific proteins to *Nodularia spumigena* CENA596 (**B**), CCY9414 (**C**), and UHCC 0039 (**D**), and *Nodularia* sp. NIES-3585 (**E**) annotated according to KEGG Orthology (KO) and SEED Subsystems, File S1: Results of the chemical analyses of the strain *N. spumigena* CENA 596. The name of variants, the method used, and the mass spectrums of nodularins, spumigins, namalides, aeruginosins, and mycosporine-like amino acids; and the results of geosmin analysis using SPME-GC-MS are shown, Table S1: Results of the antiSMASH automatic annotation of *Nodularia spumigena* CENA596, CCY9414 and UHCC 0039, and *Nodularia* sp. NIES-3585. The number of synthases and synthetases, and the biosynthetic gene cluster identified in the genomes through manual curation are shown, Table S2: Proposed function of proteins encoded by the biosynthetic gene cluster identified through manual curation in the genome of *Nodularia spumigena* strains CENA596, CCY9414, and UHCC 0039, and *Nodularia* sp. NIES-3585, Table S3: Conservation of the adenylation domain-binding pockets in genes *ndaA-D*, *spuA-B*, *aptA-D*, and *aerM-G* in the genome of *Nodularia spumigena* strains CENA596, CCY9414, and UHCC 0039; and other cyanobacteria.
