# Supplementary material for: Genomic and Metabolomic Analyses of Natural Products in Nodularia spumigena Isolated from a Shrimp Culture Pond
Source: Toxins (Basel). 2020 Feb 25;12(3):141. doi: 10.3390/toxins12030141 (PMC7150779; doi:10.3390/toxins12030141)
Supplement: Supplementary file 1 [file toxins-12-00141-s001.zip › SuppFiles_Nodularia/FileS1.pdf]

Table S4: Natural products identified in *Nodularia spumigena* CENA596

Compound	Formula	Observed *m/z*	Calculated *m/z*	Error (ppm)
Shinorine	$C_{13}H_{21}N_2O_8$	333.1277	333.1292	-4.5
Porphyra 334	$C_{14}H_{23}N_2O_8$	347.1450	347.1449	0.2
Spumigin D	$C_{29}H_{47}N_2O_{11}$	599.3187	599.3174	2.1
Spumigin F	$C_{30}H_{41}N_6O_7$	597.3030	597.3031	-0.1
Aeruginosin NOL3	$C_{30}H_{49}N_6O_6$	589.3706	589.3708	-0.3
Aeruginosin NAL2	$C_{30}H_{47}N_6O_6$	587.3551	587.3552	-0.1
Namalide C	$C_{28}H_{44}N_5O_7$	562.3225	562.3235	-1.9
Namalide B	$C_{29}H_{46}N_5O_7$	576.3384	576.3392	-1.3
Nodularin [D-Asp[1]]	$C_{40}H_{59}N_8O_{10}$	811.4346	811.4349	-0.3
Nodularin	$C_{41}H_{61}N_8O_{10}$	825.4503	825.4505	-0.2

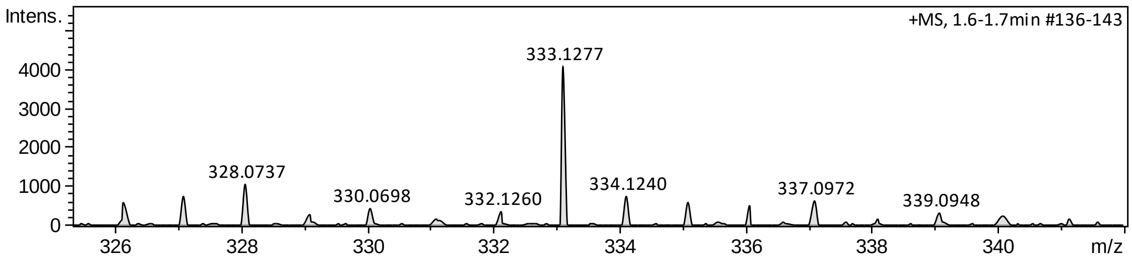

Figure S2: ESI-HR-MS spectrum of Shinorine.

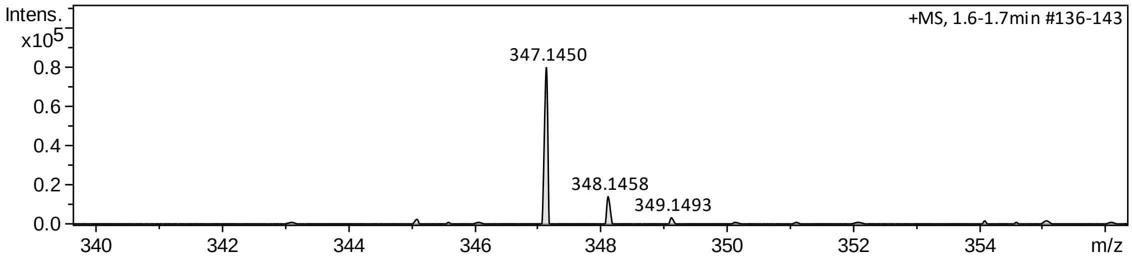

Figure S3: ESI-HR-MS spectrum of Porphyra 334.

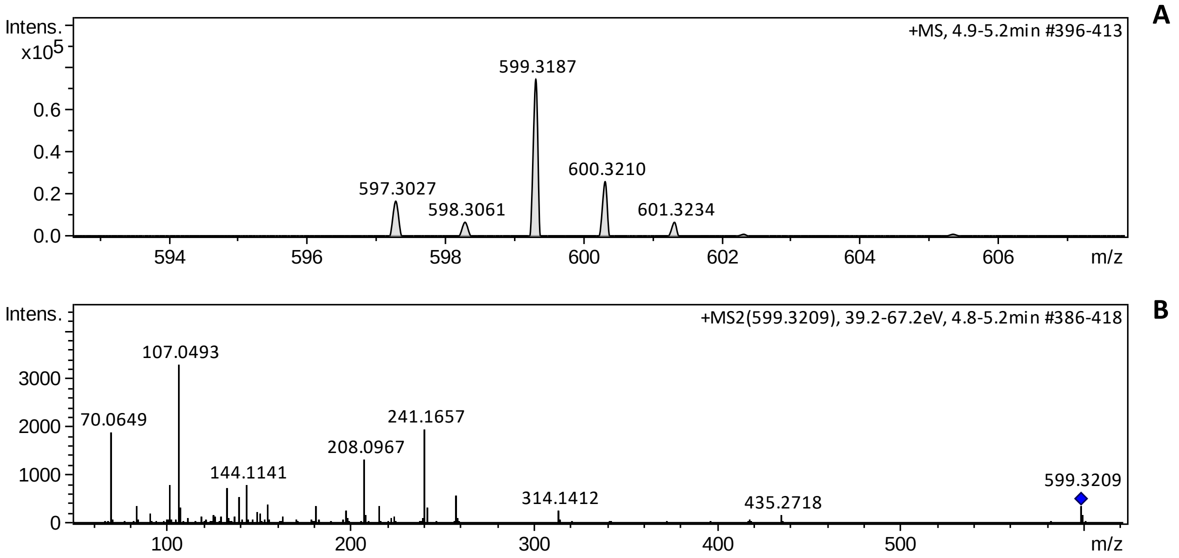

Figure S4: ESI-HR-MS (A) and MS/MS (B) spectra of Spumigin D.

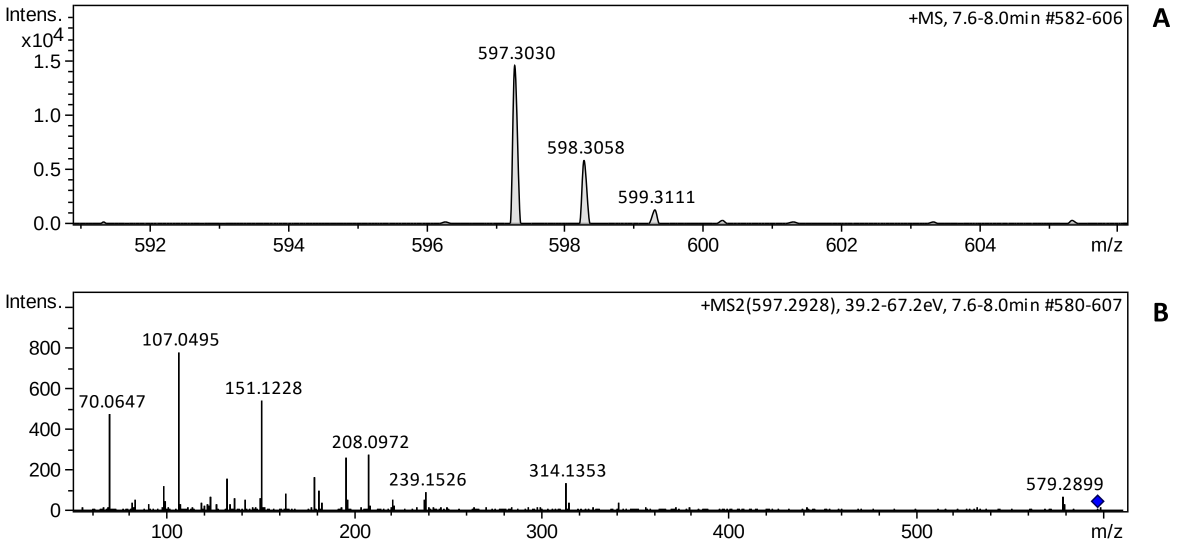

Figure S4: ESI-HR-MS (A) and MS/MS (B) spectra of Spumigin F.

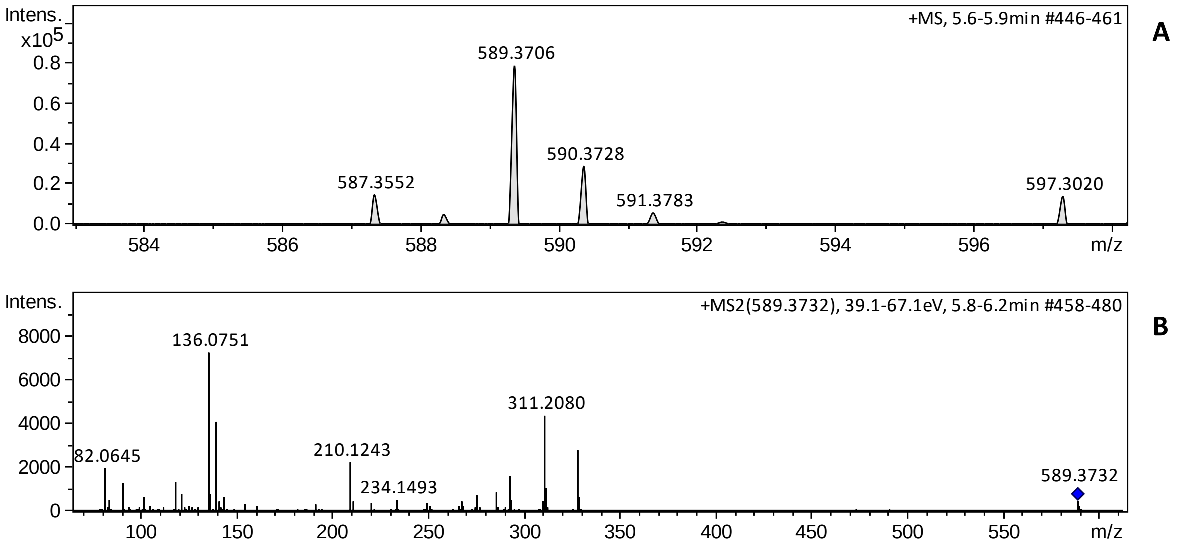

Figure S5: ESI-HR-MS (A) and MS/MS (B) spectra of Aeruginosin NOL3.

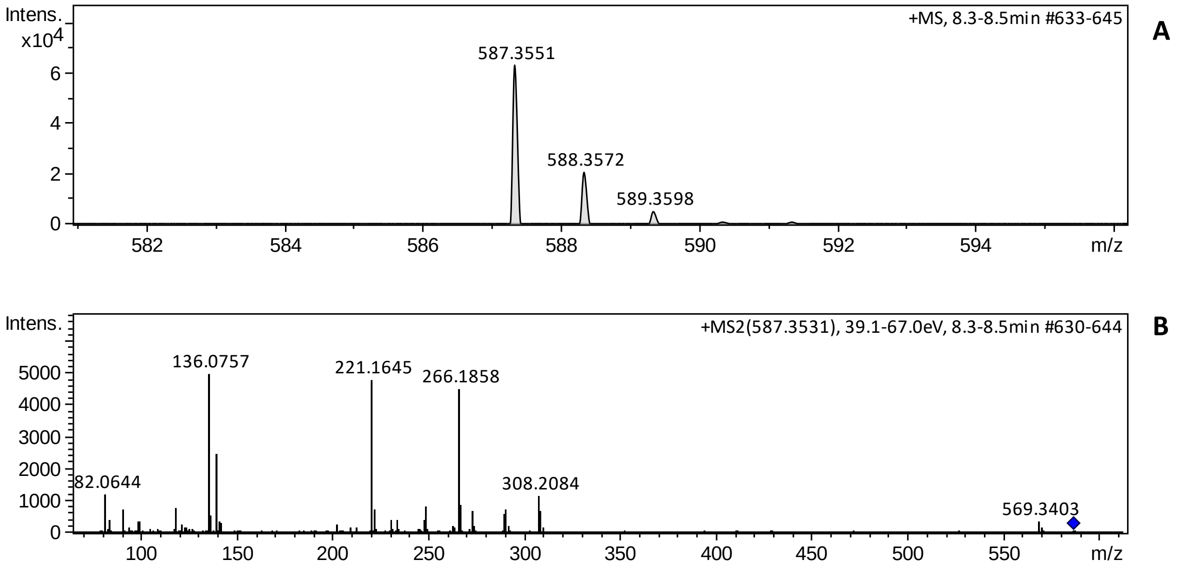

Figure S6: ESI-HR-MS (A) and MS/MS (B) spectra of Aeruginosin NAL2.

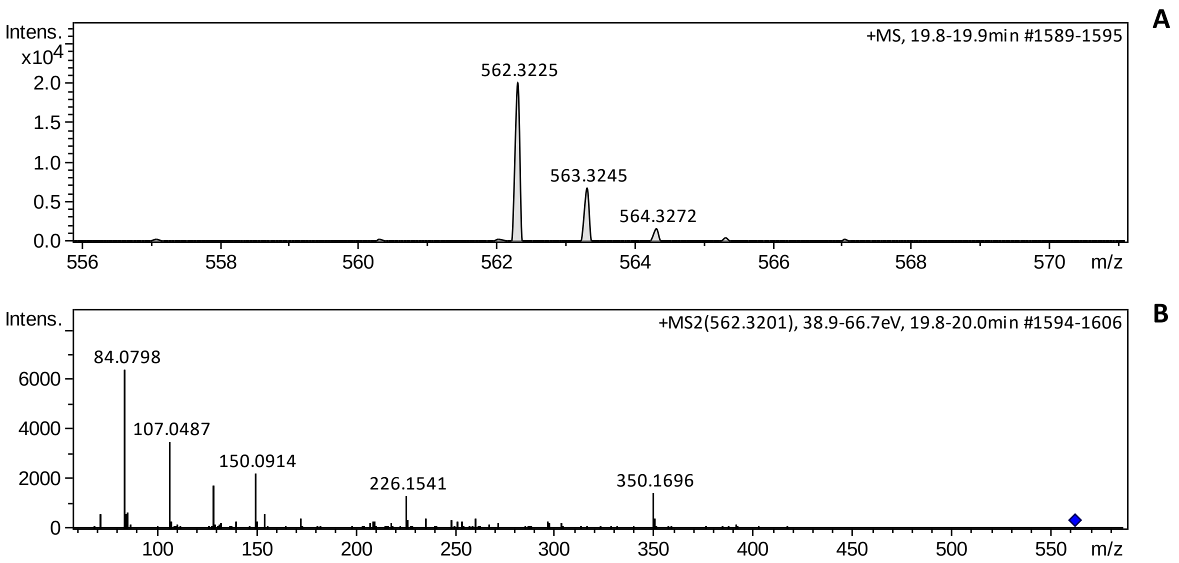

Figure S7: ESI-HR-MS (A) and MS/MS (B) spectra of Namalide C.

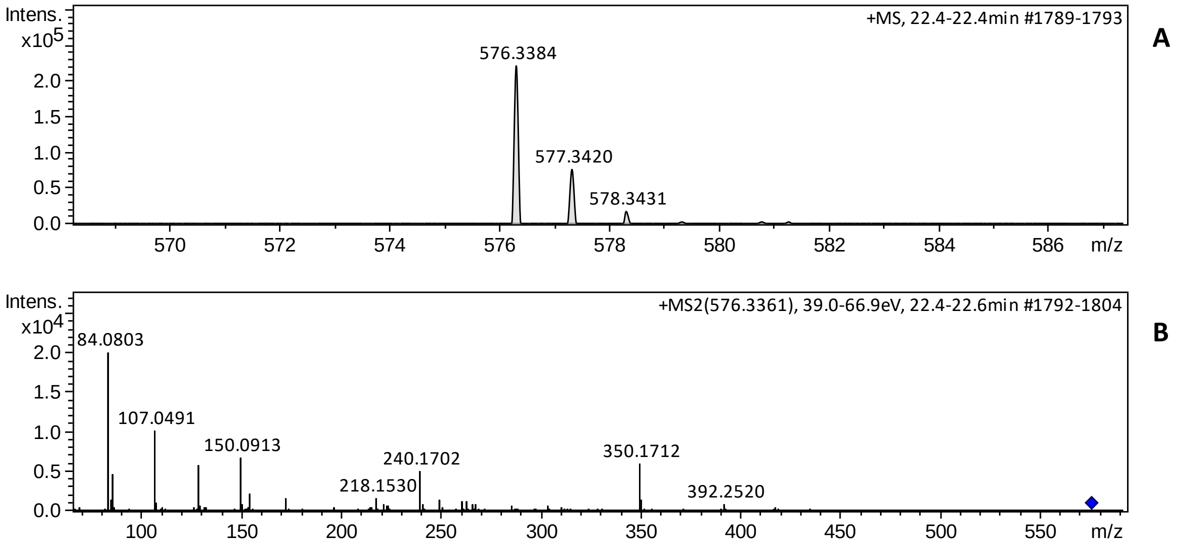

Figure S8: ESI-HR-MS (A) and MS/MS (B) spectra of Namalide B.

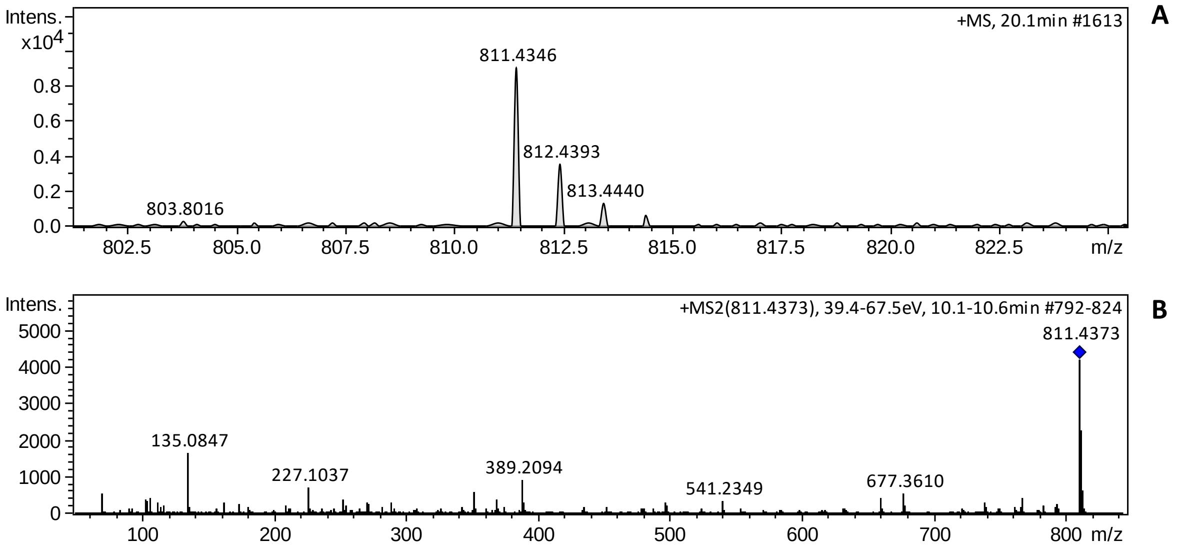

Figure S9: ESI-HR-MS (A) and MS/MS (B) spectra of Nodularin [D-Asp[1]].

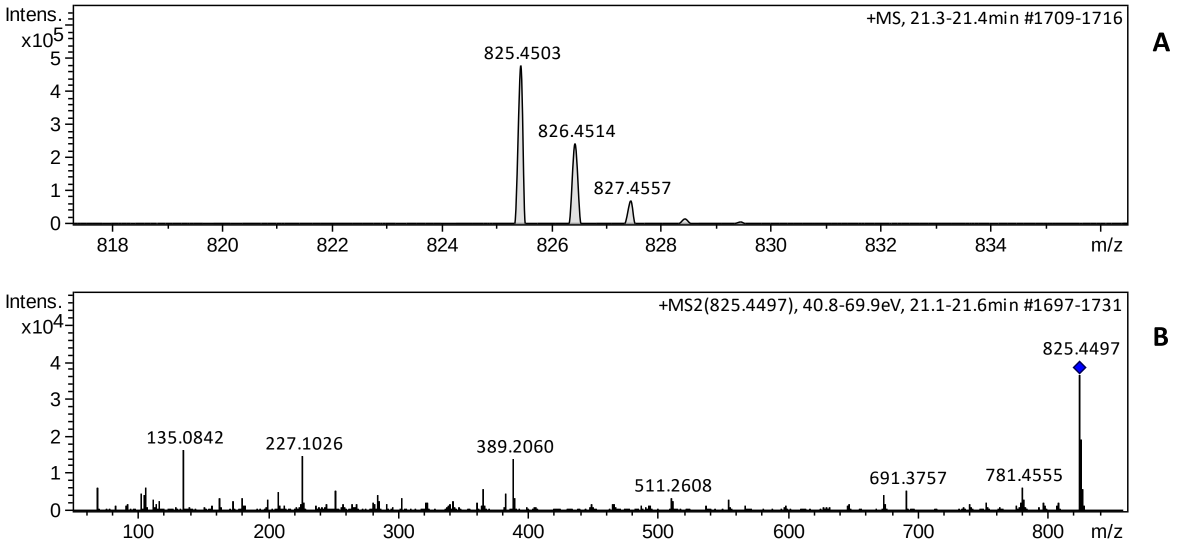

Figure S10: ESI-HR-MS (A) and MS/MS (B) spectra of Nodularin.

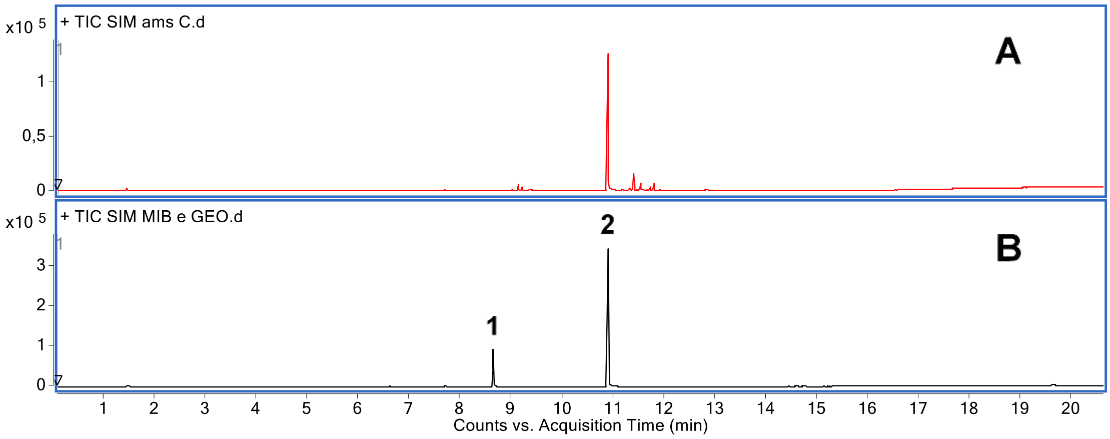

Figure S11: SPME-GC-MS analysis of CENA596. (A) fresh culture; (B) standards of methylisoborneol (1) and geosmin (2).
